# Supplementary material for: The effects of “Fangcang, Huoshenshan, and Leishenshan” hospitals and environmental factors on the mortality of COVID-19
Source: PeerJ. 2020 Jul 21;8:e9578. doi: 10.7717/peerj.9578 (PMC7380280; doi:10.7717/peerj.9578)
Supplement: Supplemental Information 1 [file peerj-08-9578-s001.docx]

1. http://www.tianqihoubao.com/lishi/wuhan/month/202001.html

2. http://www.tianqihoubao.com/lishi/wuhan/month/202002.html

3. http://www.tianqihoubao.com/lishi/huangshi/month/202001.html

4. http://www.tianqihoubao.com/lishi/huangshi/month/202002.html

5. http://www.tianqihoubao.com/lishi/shiyan/month/202001.html

6. http://www.tianqihoubao.com/lishi/shiyan/month/202002.html

7. http://www.tianqihoubao.com/lishi/yichang/month/202001.html

8. http://www.tianqihoubao.com/lishi/yichang/month/202002.html

9. http://www.tianqihoubao.com/lishi/xiangyang/month/202001.html

10. http://www.tianqihoubao.com/lishi/xiangyang/month/202002.html

11. http://www.tianqihoubao.com/lishi/ezhou/month/202001.html

12. http://www.tianqihoubao.com/lishi/ezhou/month/202002.html

13. http://www.tianqihoubao.com/lishi/jingmen/month/202001.html

14. http://www.tianqihoubao.com/lishi/jingmen/month/202002.html

15. http://www.tianqihoubao.com/lishi/xiaogan/month/202001.html

16. http://www.tianqihoubao.com/lishi/xiaogan/month/202002.html

17. http://www.tianqihoubao.com/lishi/jingzhou/month/202001.html

18. http://www.tianqihoubao.com/lishi/jingzhou/month/202002.html

19. http://www.tianqihoubao.com/lishi/huanggang/month/202001.html

20. http://www.tianqihoubao.com/lishi/huanggang/month/202002.html

21. http://www.tianqihoubao.com/lishi/xianning/month/202001.html

22. http://www.tianqihoubao.com/lishi/xianning/month/202002.html

23. http://www.tianqihoubao.com/lishi/suizhou/month/202001.html

24. http://www.tianqihoubao.com/lishi/suizhou/month/202002.html

25. http://www.tianqihoubao.com/lishi/enshi/month/202001.html

26. http://www.tianqihoubao.com/lishi/enshi/month/202002.html

27. http://www.tianqihoubao.com/lishi/xiantao/month/202001.html

28. http://www.tianqihoubao.com/lishi/xiantao/month/202002.html

29. http://www.tianqihoubao.com/lishi/qianjiang/month/202001.html

30. http://www.tianqihoubao.com/lishi/qianjiang/month/202002.html

31. http://www.tianqihoubao.com/lishi/tianmen/month/202001.html

32. http://www.tianqihoubao.com/lishi/tianmen/month/202002.html

33. http://www.tianqihoubao.com/lishi/shennongjia/month/202001.html

34. http://www.tianqihoubao.com/lishi/shennongjia/month/202002.html

35. https://m.tianqi.com/tianqi/wuhan/20200121.html

36. https://www.tianqi.com/tianqi/wuhan/20200122.html

37. https://www.tianqi.com/tianqi/wuhan/20200123.html

38. https://www.tianqi.com/tianqi/wuhan/20200124.html

39. https://www.tianqi.com/tianqi/wuhan/20200125.html

40. https://www.tianqi.com/tianqi/wuhan/20200126.html

41. https://www.tianqi.com/tianqi/wuhan/20200127.html

42. https://www.tianqi.com/tianqi/wuhan/20200128.html

43. https://www.tianqi.com/tianqi/wuhan/20200129.html

44. https://www.tianqi.com/tianqi/wuhan/20200130.html

45. https://www.tianqi.com/tianqi/wuhan/20200131.html

46. https://www.tianqi.com/tianqi/wuhan/20200201.html

47. https://www.tianqi.com/tianqi/wuhan/20200202.html

48. https://www.tianqi.com/tianqi/wuhan/20200203.html

49. https://www.tianqi.com/tianqi/wuhan/20200204.html

50. https://www.tianqi.com/tianqi/wuhan/20200205.html

51. https://www.tianqi.com/tianqi/wuhan/20200206.html

52. https://www.tianqi.com/tianqi/wuhan/20200207.html

53. https://www.tianqi.com/tianqi/wuhan/20200208.html

54. https://www.tianqi.com/tianqi/wuhan/20200209.html

55. https://www.tianqi.com/tianqi/wuhan/20200210.html

56. https://www.tianqi.com/tianqi/wuhan/20200211.html

57. https://www.tianqi.com/tianqi/wuhan/20200212.html

58. https://www.tianqi.com/tianqi/wuhan/20200213.html

59. https://www.tianqi.com/tianqi/wuhan/20200214.html

60. https://www.tianqi.com/tianqi/wuhan/20200215.html

61. https://www.tianqi.com/tianqi/wuhan/20200216.html

62. https://www.tianqi.com/tianqi/wuhan/20200217.html

63. https://www.tianqi.com/tianqi/wuhan/20200218.html

64. https://www.tianqi.com/tianqi/wuhan/20200219.html

65. https://www.tianqi.com/tianqi/wuhan/20200220.html

66. https://www.tianqi.com/tianqi/wuhan/20200221.html

67. https://m.tianqi.com/tianqi/huangshi/20200121.html

68. https://www.tianqi.com/tianqi/huangshi/20200122.html

69. https://www.tianqi.com/tianqi/huangshi/20200123.html

70. https://www.tianqi.com/tianqi/huangshi/20200124.html

71. https://www.tianqi.com/tianqi/huangshi/20200125.html

72. https://www.tianqi.com/tianqi/huangshi/20200126.html

73. https://www.tianqi.com/tianqi/huangshi/20200127.html

74. https://www.tianqi.com/tianqi/huangshi/20200128.html

75. https://www.tianqi.com/tianqi/huangshi/20200129.html

76. https://www.tianqi.com/tianqi/huangshi/20200130.html

77. https://www.tianqi.com/tianqi/huangshi/20200131.html

78. https://www.tianqi.com/tianqi/huangshi/20200201.html

79. https://www.tianqi.com/tianqi/huangshi/20200202.html

80. https://www.tianqi.com/tianqi/huangshi/20200203.html

81. https://www.tianqi.com/tianqi/huangshi/20200204.html

82. https://www.tianqi.com/tianqi/huangshi/20200205.html

83. https://www.tianqi.com/tianqi/huangshi/20200206.html

84. https://www.tianqi.com/tianqi/huangshi/20200207.html

85. https://www.tianqi.com/tianqi/huangshi/20200208.html

86. https://www.tianqi.com/tianqi/huangshi/20200209.html

87. https://www.tianqi.com/tianqi/huangshi/20200210.html

88. https://www.tianqi.com/tianqi/huangshi/20200211.html

89. https://www.tianqi.com/tianqi/huangshi/20200212.html

90. https://www.tianqi.com/tianqi/huangshi/20200213.html

91. https://www.tianqi.com/tianqi/huangshi/20200214.html

92. https://www.tianqi.com/tianqi/huangshi/20200215.html

93. https://www.tianqi.com/tianqi/huangshi/20200216.html

94. https://www.tianqi.com/tianqi/huangshi/20200217.html

95. https://www.tianqi.com/tianqi/huangshi/20200218.html

96. https://www.tianqi.com/tianqi/huangshi/20200219.html

97. https://www.tianqi.com/tianqi/huangshi/20200220.html

98. https://www.tianqi.com/tianqi/huangshi/20200221.html

99. https://m.tianqi.com/tianqi/shiyan/20200121.html

100. https://www.tianqi.com/tianqi/shiyan/20200122.html

101. https://www.tianqi.com/tianqi/shiyan/20200123.html

102. https://www.tianqi.com/tianqi/shiyan/20200124.html

103. https://www.tianqi.com/tianqi/shiyan/20200125.html

104. https://www.tianqi.com/tianqi/shiyan/20200126.html

105. https://www.tianqi.com/tianqi/shiyan/20200127.html

106. https://www.tianqi.com/tianqi/shiyan/20200128.html

107. https://www.tianqi.com/tianqi/shiyan/20200129.html

108. https://www.tianqi.com/tianqi/shiyan/20200130.html

109. https://www.tianqi.com/tianqi/shiyan/20200131.html

110. https://www.tianqi.com/tianqi/shiyan/20200201.html

111. https://www.tianqi.com/tianqi/shiyan/20200202.html

112. https://www.tianqi.com/tianqi/shiyan/20200203.html

113. https://www.tianqi.com/tianqi/shiyan/20200204.html

114. https://www.tianqi.com/tianqi/shiyan/20200205.html

115. https://www.tianqi.com/tianqi/shiyan/20200206.html

116. https://www.tianqi.com/tianqi/shiyan/20200207.html

117. https://www.tianqi.com/tianqi/shiyan/20200208.html

118. https://www.tianqi.com/tianqi/shiyan/20200209.html

119. https://www.tianqi.com/tianqi/shiyan/20200210.html

120. https://www.tianqi.com/tianqi/shiyan/20200211.html

121. https://www.tianqi.com/tianqi/shiyan/20200212.html

122. https://www.tianqi.com/tianqi/shiyan/20200213.html

123. https://www.tianqi.com/tianqi/shiyan/20200214.html

124. https://www.tianqi.com/tianqi/shiyan/20200215.html

125. https://www.tianqi.com/tianqi/shiyan/20200216.html

126. https://www.tianqi.com/tianqi/shiyan/20200217.html

127. https://www.tianqi.com/tianqi/shiyan/20200218.html

128. https://www.tianqi.com/tianqi/shiyan/20200219.html

129. https://www.tianqi.com/tianqi/shiyan/20200220.html

130. https://www.tianqi.com/tianqi/shiyan/20200221.html

131. https://m.tianqi.com/tianqi/yichang/20200121.html

132. https://www.tianqi.com/tianqi/yichang/20200122.html

133. https://www.tianqi.com/tianqi/yichang/20200123.html

134. https://www.tianqi.com/tianqi/yichang/20200124.html

135. https://www.tianqi.com/tianqi/yichang/20200125.html

136. https://www.tianqi.com/tianqi/yichang/20200126.html

137. https://www.tianqi.com/tianqi/yichang/20200127.html

138. https://www.tianqi.com/tianqi/yichang/20200128.html

139. https://www.tianqi.com/tianqi/yichang/20200129.html

140. https://www.tianqi.com/tianqi/yichang/20200130.html

141. https://www.tianqi.com/tianqi/yichang/20200131.html

142. https://www.tianqi.com/tianqi/yichang/20200201.html

143. https://www.tianqi.com/tianqi/yichang/20200202.html

144. https://www.tianqi.com/tianqi/yichang/20200203.html

145. https://www.tianqi.com/tianqi/yichang/20200204.html

146. https://www.tianqi.com/tianqi/yichang/20200205.html

147. https://www.tianqi.com/tianqi/yichang/20200206.html

148. https://www.tianqi.com/tianqi/yichang/20200207.html

149. https://www.tianqi.com/tianqi/yichang/20200208.html

150. https://www.tianqi.com/tianqi/yichang/20200209.html

151. https://www.tianqi.com/tianqi/yichang/20200210.html

152. https://www.tianqi.com/tianqi/yichang/20200211.html

153. https://www.tianqi.com/tianqi/yichang/20200212.html

154. https://www.tianqi.com/tianqi/yichang/20200213.html

155. https://www.tianqi.com/tianqi/yichang/20200214.html

156. https://www.tianqi.com/tianqi/yichang/20200215.html

157. https://www.tianqi.com/tianqi/yichang/20200216.html

158. https://www.tianqi.com/tianqi/yichang/20200217.html

159. https://www.tianqi.com/tianqi/yichang/20200218.html

160. https://www.tianqi.com/tianqi/yichang/20200219.html

161. https://www.tianqi.com/tianqi/yichang/20200220.html

162. https://www.tianqi.com/tianqi/yichang/20200221.html

163. https://m.tianqi.com/tianqi/xiangyang/20200121.html

164. https://www.tianqi.com/tianqi/xiangyang/20200122.html

165. https://www.tianqi.com/tianqi/xiangyang/20200123.html

166. https://www.tianqi.com/tianqi/xiangyang/20200124.html

167. https://www.tianqi.com/tianqi/xiangyang/20200125.html

168. https://www.tianqi.com/tianqi/xiangyang/20200126.html

169. https://www.tianqi.com/tianqi/xiangyang/20200127.html

170. https://www.tianqi.com/tianqi/xiangyang/20200128.html

171. https://www.tianqi.com/tianqi/xiangyang/20200129.html

172. https://www.tianqi.com/tianqi/xiangyang/20200130.html

173. https://www.tianqi.com/tianqi/xiangyang/20200131.html

174. https://www.tianqi.com/tianqi/xiangyang/20200201.html

175. https://www.tianqi.com/tianqi/xiangyang/20200202.html

176. https://www.tianqi.com/tianqi/xiangyang/20200203.html

177. https://www.tianqi.com/tianqi/xiangyang/20200204.html

178. https://www.tianqi.com/tianqi/xiangyang/20200205.html

179. https://www.tianqi.com/tianqi/xiangyang/20200206.html

180. https://www.tianqi.com/tianqi/xiangyang/20200207.html

181. https://www.tianqi.com/tianqi/xiangyang/20200208.html

182. https://www.tianqi.com/tianqi/xiangyang/20200209.html

183. https://www.tianqi.com/tianqi/xiangyang/20200210.html

184. https://www.tianqi.com/tianqi/xiangyang/20200211.html

185. https://www.tianqi.com/tianqi/xiangyang/20200212.html

186. https://www.tianqi.com/tianqi/xiangyang/20200213.html

187. https://www.tianqi.com/tianqi/xiangyang/20200214.html

188. https://www.tianqi.com/tianqi/xiangyang/20200215.html

189. https://www.tianqi.com/tianqi/xiangyang/20200216.html

190. https://www.tianqi.com/tianqi/xiangyang/20200217.html

191. https://www.tianqi.com/tianqi/xiangyang/20200218.html

192. https://www.tianqi.com/tianqi/xiangyang/20200219.html

193. https://www.tianqi.com/tianqi/xiangyang/20200220.html

194. https://www.tianqi.com/tianqi/xiangyang/20200221.html

195. https://m.tianqi.com/tianqi/ezhou/20200121.html

196. https://www.tianqi.com/tianqi/ezhou/20200122.html

197. https://www.tianqi.com/tianqi/ezhou/20200123.html

198. https://www.tianqi.com/tianqi/ezhou/20200124.html

199. https://www.tianqi.com/tianqi/ezhou/20200125.html

200. https://www.tianqi.com/tianqi/ezhou/20200126.html

201. https://www.tianqi.com/tianqi/ezhou/20200127.html

202. https://www.tianqi.com/tianqi/ezhou/20200128.html

203. https://www.tianqi.com/tianqi/ezhou/20200129.html

204. https://www.tianqi.com/tianqi/ezhou/20200130.html

205. https://www.tianqi.com/tianqi/ezhou/20200131.html

206. https://www.tianqi.com/tianqi/ezhou/20200201.html

207. https://www.tianqi.com/tianqi/ezhou/20200202.html

208. https://www.tianqi.com/tianqi/ezhou/20200203.html

209. https://www.tianqi.com/tianqi/ezhou/20200204.html

210. https://www.tianqi.com/tianqi/ezhou/20200205.html

211. https://www.tianqi.com/tianqi/ezhou/20200206.html

212. https://www.tianqi.com/tianqi/ezhou/20200207.html

213. https://www.tianqi.com/tianqi/ezhou/20200208.html

214. https://www.tianqi.com/tianqi/ezhou/20200209.html

215. https://www.tianqi.com/tianqi/ezhou/20200210.html

216. https://www.tianqi.com/tianqi/ezhou/20200211.html

217. https://www.tianqi.com/tianqi/ezhou/20200212.html

218. https://www.tianqi.com/tianqi/ezhou/20200213.html

219. https://www.tianqi.com/tianqi/ezhou/20200214.html

220. https://www.tianqi.com/tianqi/ezhou/20200215.html

221. https://www.tianqi.com/tianqi/ezhou/20200216.html

222. https://www.tianqi.com/tianqi/ezhou/20200217.html

223. https://www.tianqi.com/tianqi/ezhou/20200218.html

224. https://www.tianqi.com/tianqi/ezhou/20200219.html

225. https://www.tianqi.com/tianqi/ezhou/20200220.html

226. https://www.tianqi.com/tianqi/ezhou/20200221.html

227. https://m.tianqi.com/tianqi/jingmen/20200121.html

228. https://www.tianqi.com/tianqi/jingmen/20200122.html

229. https://www.tianqi.com/tianqi/jingmen/20200123.html

230. https://www.tianqi.com/tianqi/jingmen/20200124.html

231. https://www.tianqi.com/tianqi/jingmen/20200125.html

232. https://www.tianqi.com/tianqi/jingmen/20200126.html

233. https://www.tianqi.com/tianqi/jingmen/20200127.html

234. https://www.tianqi.com/tianqi/jingmen/20200128.html

235. https://www.tianqi.com/tianqi/jingmen/20200129.html

236. https://www.tianqi.com/tianqi/jingmen/20200130.html

237. https://www.tianqi.com/tianqi/jingmen/20200131.html

238. https://www.tianqi.com/tianqi/jingmen/20200201.html

239. https://www.tianqi.com/tianqi/jingmen/20200202.html

240. https://www.tianqi.com/tianqi/jingmen/20200203.html

241. https://www.tianqi.com/tianqi/jingmen/20200204.html

242. https://www.tianqi.com/tianqi/jingmen/20200205.html

243. https://www.tianqi.com/tianqi/jingmen/20200206.html

244. https://www.tianqi.com/tianqi/jingmen/20200207.html

245. https://www.tianqi.com/tianqi/jingmen/20200208.html

246. https://www.tianqi.com/tianqi/jingmen/20200209.html

247. https://www.tianqi.com/tianqi/jingmen/20200210.html

248. https://www.tianqi.com/tianqi/jingmen/20200211.html

249. https://www.tianqi.com/tianqi/jingmen/20200212.html

250. https://www.tianqi.com/tianqi/jingmen/20200213.html

251. https://www.tianqi.com/tianqi/jingmen/20200214.html

252. https://www.tianqi.com/tianqi/jingmen/20200215.html

253. https://www.tianqi.com/tianqi/jingmen/20200216.html

254. https://www.tianqi.com/tianqi/jingmen/20200217.html

255. https://www.tianqi.com/tianqi/jingmen/20200218.html

256. https://www.tianqi.com/tianqi/jingmen/20200219.html

257. https://www.tianqi.com/tianqi/jingmen/20200220.html

258. https://www.tianqi.com/tianqi/jingmen/20200221.html

259. https://m.tianqi.com/tianqi/xiaogan/20200121.html

260. https://www.tianqi.com/tianqi/xiaogan/20200122.html

261. https://www.tianqi.com/tianqi/xiaogan/20200123.html

262. https://www.tianqi.com/tianqi/xiaogan/20200124.html

263. https://www.tianqi.com/tianqi/xiaogan/20200125.html

264. https://www.tianqi.com/tianqi/xiaogan/20200126.html

265. https://www.tianqi.com/tianqi/xiaogan/20200127.html

266. https://www.tianqi.com/tianqi/xiaogan/20200128.html

267. https://www.tianqi.com/tianqi/xiaogan/20200129.html

268. https://www.tianqi.com/tianqi/xiaogan/20200130.html

269. https://www.tianqi.com/tianqi/xiaogan/20200131.html

270. https://www.tianqi.com/tianqi/xiaogan/20200201.html

271. https://www.tianqi.com/tianqi/xiaogan/20200202.html

272. https://www.tianqi.com/tianqi/xiaogan/20200203.html

273. https://www.tianqi.com/tianqi/xiaogan/20200204.html

274. https://www.tianqi.com/tianqi/xiaogan/20200205.html

275. https://www.tianqi.com/tianqi/xiaogan/20200206.html

276. https://www.tianqi.com/tianqi/xiaogan/20200207.html

277. https://www.tianqi.com/tianqi/xiaogan/20200208.html

278. https://www.tianqi.com/tianqi/xiaogan/20200209.html

279. https://www.tianqi.com/tianqi/xiaogan/20200210.html

280. https://www.tianqi.com/tianqi/xiaogan/20200211.html

281. https://www.tianqi.com/tianqi/xiaogan/20200212.html

282. https://www.tianqi.com/tianqi/xiaogan/20200213.html

283. https://www.tianqi.com/tianqi/xiaogan/20200214.html

284. https://www.tianqi.com/tianqi/xiaogan/20200215.html

285. https://www.tianqi.com/tianqi/xiaogan/20200216.html

286. https://www.tianqi.com/tianqi/xiaogan/20200217.html

287. https://www.tianqi.com/tianqi/xiaogan/20200218.html

288. https://www.tianqi.com/tianqi/xiaogan/20200219.html

289. https://www.tianqi.com/tianqi/xiaogan/20200220.html

290. https://www.tianqi.com/tianqi/xiaogan/20200221.html

291. https://m.tianqi.com/tianqi/jingzhou/20200121.html

292. https://www.tianqi.com/tianqi/jingzhou/20200122.html

293. https://www.tianqi.com/tianqi/jingzhou/20200123.html

294. https://www.tianqi.com/tianqi/jingzhou/20200124.html

295. https://www.tianqi.com/tianqi/jingzhou/20200125.html

296. https://www.tianqi.com/tianqi/jingzhou/20200126.html

297. https://www.tianqi.com/tianqi/jingzhou/20200127.html

298. https://www.tianqi.com/tianqi/jingzhou/20200128.html

299. https://www.tianqi.com/tianqi/jingzhou/20200129.html

300. https://www.tianqi.com/tianqi/jingzhou/20200130.html

301. https://www.tianqi.com/tianqi/jingzhou/20200131.html

302. https://www.tianqi.com/tianqi/jingzhou/20200201.html

303. https://www.tianqi.com/tianqi/jingzhou/20200202.html

304. https://www.tianqi.com/tianqi/jingzhou/20200203.html

305. https://www.tianqi.com/tianqi/jingzhou/20200204.html

306. https://www.tianqi.com/tianqi/jingzhou/20200205.html

307. https://www.tianqi.com/tianqi/jingzhou/20200206.html

308. https://www.tianqi.com/tianqi/jingzhou/20200207.html

309. https://www.tianqi.com/tianqi/jingzhou/20200208.html

310. https://www.tianqi.com/tianqi/jingzhou/20200209.html

311. https://www.tianqi.com/tianqi/jingzhou/20200210.html

312. https://www.tianqi.com/tianqi/jingzhou/20200211.html

313. https://www.tianqi.com/tianqi/jingzhou/20200212.html

314. https://www.tianqi.com/tianqi/jingzhou/20200213.html

315. https://www.tianqi.com/tianqi/jingzhou/20200214.html

316. https://www.tianqi.com/tianqi/jingzhou/20200215.html

317. https://www.tianqi.com/tianqi/jingzhou/20200216.html

318. https://www.tianqi.com/tianqi/jingzhou/20200217.html

319. https://www.tianqi.com/tianqi/jingzhou/20200218.html

320. https://www.tianqi.com/tianqi/jingzhou/20200219.html

321. https://www.tianqi.com/tianqi/jingzhou/20200220.html

322. https://www.tianqi.com/tianqi/jingzhou/20200221.html

323. https://m.tianqi.com/tianqi/huanggang/20200121.html

324. https://www.tianqi.com/tianqi/huanggang/20200122.html

325. https://www.tianqi.com/tianqi/huanggang/20200123.html

326. https://www.tianqi.com/tianqi/huanggang/20200124.html

327. https://www.tianqi.com/tianqi/huanggang/20200125.html

328. https://www.tianqi.com/tianqi/huanggang/20200126.html

329. https://www.tianqi.com/tianqi/huanggang/20200127.html

330. https://www.tianqi.com/tianqi/huanggang/20200128.html

331. https://www.tianqi.com/tianqi/huanggang/20200129.html

332. https://www.tianqi.com/tianqi/huanggang/20200130.html

333. https://www.tianqi.com/tianqi/huanggang/20200131.html

334. https://www.tianqi.com/tianqi/huanggang/20200201.html

335. https://www.tianqi.com/tianqi/huanggang/20200202.html

336. https://www.tianqi.com/tianqi/huanggang/20200203.html

337. https://www.tianqi.com/tianqi/huanggang/20200204.html

338. https://www.tianqi.com/tianqi/huanggang/20200205.html

339. https://www.tianqi.com/tianqi/huanggang/20200206.html

340. https://www.tianqi.com/tianqi/huanggang/20200207.html

341. https://www.tianqi.com/tianqi/huanggang/20200208.html

342. https://www.tianqi.com/tianqi/huanggang/20200209.html

343. https://www.tianqi.com/tianqi/huanggang/20200210.html

344. https://www.tianqi.com/tianqi/huanggang/20200211.html

345. https://www.tianqi.com/tianqi/huanggang/20200212.html

346. https://www.tianqi.com/tianqi/huanggang/20200213.html

347. https://www.tianqi.com/tianqi/huanggang/20200214.html

348. https://www.tianqi.com/tianqi/huanggang/20200215.html

349. https://www.tianqi.com/tianqi/huanggang/20200216.html

350. https://www.tianqi.com/tianqi/huanggang/20200217.html

351. https://www.tianqi.com/tianqi/huanggang/20200218.html

352. https://www.tianqi.com/tianqi/huanggang/20200219.html

353. https://www.tianqi.com/tianqi/huanggang/20200220.html

354. https://www.tianqi.com/tianqi/huanggang/20200221.html

355. https://m.tianqi.com/tianqi/xianning/20200121.html

356. https://www.tianqi.com/tianqi/xianning/20200122.html

357. https://www.tianqi.com/tianqi/xianning/20200123.html

358. https://www.tianqi.com/tianqi/xianning/20200124.html

359. https://www.tianqi.com/tianqi/xianning/20200125.html

360. https://www.tianqi.com/tianqi/xianning/20200126.html

361. https://www.tianqi.com/tianqi/xianning/20200127.html

362. https://www.tianqi.com/tianqi/xianning/20200128.html

363. https://www.tianqi.com/tianqi/xianning/20200129.html

364. https://www.tianqi.com/tianqi/xianning/20200130.html

365. https://www.tianqi.com/tianqi/xianning/20200131.html

366. https://www.tianqi.com/tianqi/xianning/20200201.html

367. https://www.tianqi.com/tianqi/xianning/20200202.html

368. https://www.tianqi.com/tianqi/xianning/20200203.html

369. https://www.tianqi.com/tianqi/xianning/20200204.html

370. https://www.tianqi.com/tianqi/xianning/20200205.html

371. https://www.tianqi.com/tianqi/xianning/20200206.html

372. https://www.tianqi.com/tianqi/xianning/20200207.html

373. https://www.tianqi.com/tianqi/xianning/20200208.html

374. https://www.tianqi.com/tianqi/xianning/20200209.html

375. https://www.tianqi.com/tianqi/xianning/20200210.html

376. https://www.tianqi.com/tianqi/xianning/20200211.html

377. https://www.tianqi.com/tianqi/xianning/20200212.html

378. https://www.tianqi.com/tianqi/xianning/20200213.html

379. https://www.tianqi.com/tianqi/xianning/20200214.html

380. https://www.tianqi.com/tianqi/xianning/20200215.html

381. https://www.tianqi.com/tianqi/xianning/20200216.html

382. https://www.tianqi.com/tianqi/xianning/20200217.html

383. https://www.tianqi.com/tianqi/xianning/20200218.html

384. https://www.tianqi.com/tianqi/xianning/20200219.html

385. https://www.tianqi.com/tianqi/xianning/20200220.html

386. https://www.tianqi.com/tianqi/xianning/20200221.html

387. https://m.tianqi.com/tianqi/suizhou/20200121.html

388. https://www.tianqi.com/tianqi/suizhou/20200122.html

389. https://www.tianqi.com/tianqi/suizhou/20200123.html

390. https://www.tianqi.com/tianqi/suizhou/20200124.html

391. https://www.tianqi.com/tianqi/suizhou/20200125.html

392. https://www.tianqi.com/tianqi/suizhou/20200126.html

393. https://www.tianqi.com/tianqi/suizhou/20200127.html

394. https://www.tianqi.com/tianqi/suizhou/20200128.html

395. https://www.tianqi.com/tianqi/suizhou/20200129.html

396. https://www.tianqi.com/tianqi/suizhou/20200130.html

397. https://www.tianqi.com/tianqi/suizhou/20200131.html

398. https://www.tianqi.com/tianqi/suizhou/20200201.html

399. https://www.tianqi.com/tianqi/suizhou/20200202.html

400. https://www.tianqi.com/tianqi/suizhou/20200203.html

401. https://www.tianqi.com/tianqi/suizhou/20200204.html

402. https://www.tianqi.com/tianqi/suizhou/20200205.html

403. https://www.tianqi.com/tianqi/suizhou/20200206.html

404. https://www.tianqi.com/tianqi/suizhou/20200207.html

405. https://www.tianqi.com/tianqi/suizhou/20200208.html

406. https://www.tianqi.com/tianqi/suizhou/20200209.html

407. https://www.tianqi.com/tianqi/suizhou/20200210.html

408. https://www.tianqi.com/tianqi/suizhou/20200211.html

409. https://www.tianqi.com/tianqi/suizhou/20200212.html

410. https://www.tianqi.com/tianqi/suizhou/20200213.html

411. https://www.tianqi.com/tianqi/suizhou/20200214.html

412. https://www.tianqi.com/tianqi/suizhou/20200215.html

413. https://www.tianqi.com/tianqi/suizhou/20200216.html

414. https://www.tianqi.com/tianqi/suizhou/20200217.html

415. https://www.tianqi.com/tianqi/suizhou/20200218.html

416. https://www.tianqi.com/tianqi/suizhou/20200219.html

417. https://www.tianqi.com/tianqi/suizhou/20200220.html

418. https://www.tianqi.com/tianqi/suizhou/20200221.html

419. https://m.tianqi.com/tianqi/enshi/20200121.html

420. https://www.tianqi.com/tianqi/enshi/20200122.html

421. https://www.tianqi.com/tianqi/enshi/20200123.html

422. https://www.tianqi.com/tianqi/enshi/20200124.html

423. https://www.tianqi.com/tianqi/enshi/20200125.html

424. https://www.tianqi.com/tianqi/enshi/20200126.html

425. https://www.tianqi.com/tianqi/enshi/20200127.html

426. https://www.tianqi.com/tianqi/enshi/20200128.html

427. https://www.tianqi.com/tianqi/enshi/20200129.html

428. https://www.tianqi.com/tianqi/enshi/20200130.html

429. https://www.tianqi.com/tianqi/enshi/20200131.html

430. https://www.tianqi.com/tianqi/enshi/20200201.html

431. https://www.tianqi.com/tianqi/enshi/20200202.html

432. https://www.tianqi.com/tianqi/enshi/20200203.html

433. https://www.tianqi.com/tianqi/enshi/20200204.html

434. https://www.tianqi.com/tianqi/enshi/20200205.html

435. https://www.tianqi.com/tianqi/enshi/20200206.html

436. https://www.tianqi.com/tianqi/enshi/20200207.html

437. https://www.tianqi.com/tianqi/enshi/20200208.html

438. https://www.tianqi.com/tianqi/enshi/20200209.html

439. https://www.tianqi.com/tianqi/enshi/20200210.html

440. https://www.tianqi.com/tianqi/enshi/20200211.html

441. https://www.tianqi.com/tianqi/enshi/20200212.html

442. https://www.tianqi.com/tianqi/enshi/20200213.html

443. https://www.tianqi.com/tianqi/enshi/20200214.html

444. https://www.tianqi.com/tianqi/enshi/20200215.html

445. https://www.tianqi.com/tianqi/enshi/20200216.html

446. https://www.tianqi.com/tianqi/enshi/20200217.html

447. https://www.tianqi.com/tianqi/enshi/20200218.html

448. https://www.tianqi.com/tianqi/enshi/20200219.html

449. https://www.tianqi.com/tianqi/enshi/20200220.html

450. https://www.tianqi.com/tianqi/enshi/20200221.html

451. https://m.tianqi.com/tianqi/xiantao/20200121.html

452. https://www.tianqi.com/tianqi/xiantao/20200122.html

453. https://www.tianqi.com/tianqi/xiantao/20200123.html

454. https://www.tianqi.com/tianqi/xiantao/20200124.html

455. https://www.tianqi.com/tianqi/xiantao/20200125.html

456. https://www.tianqi.com/tianqi/xiantao/20200126.html

457. https://www.tianqi.com/tianqi/xiantao/20200127.html

458. https://www.tianqi.com/tianqi/xiantao/20200128.html

459. https://www.tianqi.com/tianqi/xiantao/20200129.html

460. https://www.tianqi.com/tianqi/xiantao/20200130.html

461. https://www.tianqi.com/tianqi/xiantao/20200131.html

462. https://www.tianqi.com/tianqi/xiantao/20200201.html

463. https://www.tianqi.com/tianqi/xiantao/20200202.html

464. https://www.tianqi.com/tianqi/xiantao/20200203.html

465. https://www.tianqi.com/tianqi/xiantao/20200204.html

466. https://www.tianqi.com/tianqi/xiantao/20200205.html

467. https://www.tianqi.com/tianqi/xiantao/20200206.html

468. https://www.tianqi.com/tianqi/xiantao/20200207.html

469. https://www.tianqi.com/tianqi/xiantao/20200208.html

470. https://www.tianqi.com/tianqi/xiantao/20200209.html

471. https://www.tianqi.com/tianqi/xiantao/20200210.html

472. https://www.tianqi.com/tianqi/xiantao/20200211.html

473. https://www.tianqi.com/tianqi/xiantao/20200212.html

474. https://www.tianqi.com/tianqi/xiantao/20200213.html

475. https://www.tianqi.com/tianqi/xiantao/20200214.html

476. https://www.tianqi.com/tianqi/xiantao/20200215.html

477. https://www.tianqi.com/tianqi/xiantao/20200216.html

478. https://www.tianqi.com/tianqi/xiantao/20200217.html

479. https://www.tianqi.com/tianqi/xiantao/20200218.html

480. https://www.tianqi.com/tianqi/xiantao/20200219.html

481. https://www.tianqi.com/tianqi/xiantao/20200220.html

482. https://www.tianqi.com/tianqi/xiantao/20200221.html

483. https://m.tianqi.com/tianqi/qianjiang/20200121.html

484. https://www.tianqi.com/tianqi/qianjiang/20200122.html

485. https://www.tianqi.com/tianqi/qianjiang/20200123.html

486. https://www.tianqi.com/tianqi/qianjiang/20200124.html

487. https://www.tianqi.com/tianqi/qianjiang/20200125.html

488. https://www.tianqi.com/tianqi/qianjiang/20200126.html

489. https://www.tianqi.com/tianqi/qianjiang/20200127.html

490. https://www.tianqi.com/tianqi/qianjiang/20200128.html

491. https://www.tianqi.com/tianqi/qianjiang/20200129.html

492. https://www.tianqi.com/tianqi/qianjiang/20200130.html

493. https://www.tianqi.com/tianqi/qianjiang/20200131.html

494. https://www.tianqi.com/tianqi/qianjiang/20200201.html

495. https://www.tianqi.com/tianqi/qianjiang/20200202.html

496. https://www.tianqi.com/tianqi/qianjiang/20200203.html

497. https://www.tianqi.com/tianqi/qianjiang/20200204.html

498. https://www.tianqi.com/tianqi/qianjiang/20200205.html

499. https://www.tianqi.com/tianqi/qianjiang/20200206.html

500. https://www.tianqi.com/tianqi/qianjiang/20200207.html

501. https://www.tianqi.com/tianqi/qianjiang/20200208.html

502. https://www.tianqi.com/tianqi/qianjiang/20200209.html

503. https://www.tianqi.com/tianqi/qianjiang/20200210.html

504. https://www.tianqi.com/tianqi/qianjiang/20200211.html

505. https://www.tianqi.com/tianqi/qianjiang/20200212.html

506. https://www.tianqi.com/tianqi/qianjiang/20200213.html

507. https://www.tianqi.com/tianqi/qianjiang/20200214.html

508. https://www.tianqi.com/tianqi/qianjiang/20200215.html

509. https://www.tianqi.com/tianqi/qianjiang/20200216.html

510. https://www.tianqi.com/tianqi/qianjiang/20200217.html

511. https://www.tianqi.com/tianqi/qianjiang/20200218.html

512. https://www.tianqi.com/tianqi/qianjiang/20200219.html

513. https://www.tianqi.com/tianqi/qianjiang/20200220.html

514. https://www.tianqi.com/tianqi/qianjiang/20200221.html

515. https://m.tianqi.com/tianqi/tianmen/20200121.html

516. https://www.tianqi.com/tianqi/tianmen/20200122.html

517. https://www.tianqi.com/tianqi/tianmen/20200123.html

518. https://www.tianqi.com/tianqi/tianmen/20200124.html

519. https://www.tianqi.com/tianqi/tianmen/20200125.html

520. https://www.tianqi.com/tianqi/tianmen/20200126.html

521. https://www.tianqi.com/tianqi/tianmen/20200127.html

522. https://www.tianqi.com/tianqi/tianmen/20200128.html

523. https://www.tianqi.com/tianqi/tianmen/20200129.html

524. https://www.tianqi.com/tianqi/tianmen/20200130.html

525. https://www.tianqi.com/tianqi/tianmen/20200131.html

526. https://www.tianqi.com/tianqi/tianmen/20200201.html

527. https://www.tianqi.com/tianqi/tianmen/20200202.html

528. https://www.tianqi.com/tianqi/tianmen/20200203.html

529. https://www.tianqi.com/tianqi/tianmen/20200204.html

530. https://www.tianqi.com/tianqi/tianmen/20200205.html

531. https://www.tianqi.com/tianqi/tianmen/20200206.html

532. https://www.tianqi.com/tianqi/tianmen/20200207.html

533. https://www.tianqi.com/tianqi/tianmen/20200208.html

534. https://www.tianqi.com/tianqi/tianmen/20200209.html

535. https://www.tianqi.com/tianqi/tianmen/20200210.html

536. https://www.tianqi.com/tianqi/tianmen/20200211.html

537. https://www.tianqi.com/tianqi/tianmen/20200212.html

538. https://www.tianqi.com/tianqi/tianmen/20200213.html

539. https://www.tianqi.com/tianqi/tianmen/20200214.html

540. https://www.tianqi.com/tianqi/tianmen/20200215.html

541. https://www.tianqi.com/tianqi/tianmen/20200216.html

542. https://www.tianqi.com/tianqi/tianmen/20200217.html

543. https://www.tianqi.com/tianqi/tianmen/20200218.html

544. https://www.tianqi.com/tianqi/tianmen/20200219.html

545. https://www.tianqi.com/tianqi/tianmen/20200220.html

546. https://www.tianqi.com/tianqi/tianmen/20200221.html

547. https://m.tianqi.com/tianqi/shennongjia/20200121.html

548. https://www.tianqi.com/tianqi/shennongjia/20200122.html

549. https://www.tianqi.com/tianqi/shennongjia/20200123.html

550. https://www.tianqi.com/tianqi/shennongjia/20200124.html

551. https://www.tianqi.com/tianqi/shennongjia/20200125.html

552. https://www.tianqi.com/tianqi/shennongjia/20200126.html

553. https://www.tianqi.com/tianqi/shennongjia/20200127.html

554. https://www.tianqi.com/tianqi/shennongjia/20200128.html

555. https://www.tianqi.com/tianqi/shennongjia/20200129.html

556. https://www.tianqi.com/tianqi/shennongjia/20200130.html

557. https://www.tianqi.com/tianqi/shennongjia/20200131.html

558. https://www.tianqi.com/tianqi/shennongjia/20200201.html

559. https://www.tianqi.com/tianqi/shennongjia/20200202.html

560. https://www.tianqi.com/tianqi/shennongjia/20200203.html

561. https://www.tianqi.com/tianqi/shennongjia/20200204.html

562. https://www.tianqi.com/tianqi/shennongjia/20200205.html

563. https://www.tianqi.com/tianqi/shennongjia/20200206.html

564. https://www.tianqi.com/tianqi/shennongjia/20200207.html

565. https://www.tianqi.com/tianqi/shennongjia/20200208.html

566. https://www.tianqi.com/tianqi/shennongjia/20200209.html

567. https://www.tianqi.com/tianqi/shennongjia/20200210.html

568. https://www.tianqi.com/tianqi/shennongjia/20200211.html

569. https://www.tianqi.com/tianqi/shennongjia/20200212.html

570. https://www.tianqi.com/tianqi/shennongjia/20200213.html

571. https://www.tianqi.com/tianqi/shennongjia/20200214.html

572. https://www.tianqi.com/tianqi/shennongjia/20200215.html

573. https://www.tianqi.com/tianqi/shennongjia/20200216.html

574. https://www.tianqi.com/tianqi/shennongjia/20200217.html

575. https://www.tianqi.com/tianqi/shennongjia/20200218.html

576. https://www.tianqi.com/tianqi/shennongjia/20200219.html

577. https://www.tianqi.com/tianqi/shennongjia/20200220.html

578. https://www.tianqi.com/tianqi/shennongjia/20200221.html
